# Impact of Social Media on Patient Expectations and Satisfaction: A Qualitative Exploration in Multan, Pakistan

**DOI:** 10.7759/cureus.77838

**Published:** 2025-01-22

**Authors:** Abdul Ghafoor, Hassan Jan, Fatima Shafqat, Muhammad Asif Shahzad, Ayma Syed, Asifa Iqbal, Hafsa Naveed, Arooba Ahmad

**Affiliations:** 1 Department of Dental Surgery, Ahmad Hospital, Swat, PAK; 2 Department of Operative Dentistry, Peshawar Medical and Dental College, Peshawar, PAK; 3 Department of Physiology, The University of Lahore, Multan, PAK; 4 Department of Oral and Maxillofacial Surgery, Azra Naheed Dental College, Superior University, Lahore, PAK; 5 Department of Community and Preventive Dentistry, Azra Naheed Dental College, Superior University, Lahore, PAK; 6 Department of Oral Pathology, Rashid Latif Dental College, Lahore, PAK; 7 Department of Operative Dentistry and Endodontics, Multan Medical and Dental College, Multan, PAK; 8 Department of Oral and Maxillofacial Surgery, Bakhtawar Amin Medical and Dental College, Multan, PAK

**Keywords:** dental patient, healthcare policy, health communication, impact, pakistan, patient education, patients’ expectations, qualitative research, satisfaction, social media

## Abstract

Background: Social media (SM) has become an innovative area that is revolutionizing the process of healthcare decision-making, with deep influence on the dental specialty, where aesthetic preferences of patients create high-level expectations. Despite SM being used widely in Pakistan and its penetration rate increasing very fast, its effectiveness and its role in patient expectations and satisfaction towards dental procedures are underresearched. This research aims to explore the perspective of patients and dentists regarding the influence of social media in shaping expectations and satisfaction with dental procedures.

Methodology: This cross-sectional, exploratory qualitative study was carried out in dental clinics in Multan, Pakistan. Semi-structured interviews were conducted with 17 patients, and 10 dental consultants participated in focus group discussions (FGDs). Purposive sampling ensured the participation of a diverse range of participants. Transcripts were transcribed manually and analyzed using a thematic analysis framework.

Results: The results showed that SM greatly affects patient expectations, which resulted in unreasonable expectations from patients (70%) based on idealized images of dental procedures. Participants (47%) acknowledged the role of SM in increasing patients' knowledge and linking them to practitioners but also expressed disappointment when their expectations, especially with relation to cosmetic results-were not satisfied (65%). Unrealistic information circulated on SM and poor communication during consultations aggravated this discontent. Dentists voiced worries about ethical conundrums, increased pressure to satisfy unreasonable expectations, and the lack of organized programs meant to counter disinformation and misconceptions. To close the discrepancy between expectations and clinical reality, participants advised transparent and realistic SM content, enhanced patient communication, and public awareness initiatives. Participants also recommended that institutions and the government should offer subsidized treatment options for non-affording populations.

Conclusion: The study served as a dual lens, both enabling awareness and serving as a source of misinformation for dental treatments. The study highlighted that exaggerated and unrealistic patient expectations often led to disappointment. Patient education, improved communication strategies, and ethical use of SM were pointed out as means through which these challenges can be addressed. The study proposed that in order to utilize the maximum potential of SM in patient satisfaction and decision-making, institutional interventions, such as structured training for healthcare providers and policy-based public awareness campaigns, are necessary.

## Introduction

Social media (SM) is a flexible term with a constantly expanding definition, as many novelties are appearing in this field. Generally, it refers to all web-based resources that can facilitate the coalescing, informing, and quick communication between members of various groups, communities, and regions. People also post daily updates such as status messages, multimedia texts, pictures, and audio recordings. It is also possible for people to share their opinions and feelings freely and engage in discussions on topics they are passionate about. Moreover, SM is a powerful mode that influences not only communication but also relationships among people and companies [1]. SM, now recognized as a socio-cultural agent of change, shapes the flow of information and affects the provider-patient relationship [2].

SM has changed the way people interact, disseminate, and utilize information in society. Facebook, Instagram, Threads, and TikTok are examples of tools that have expanded access to information and have united millions of people around the globe, changing social paradigms [3]. Recent developments of such SM platforms as X and YouTube have facilitated access to knowledge and experiences, products, and services for those in the healthcare sector. In SM, patients around the globe have witnessed a shift in patient-provider relationships through information sharing, peer support, and participation [4]. However, this technological marvel also creates a new problem where dental procedures can become a subject of public discussion due to connections of aesthetics [5].

In healthcare, SM moves beyond just communication and sharing of information. The patient is becoming more connected to SM's visuals, patient stories, and experiences shared online [6]. In the field of dentistry, where aesthetics plays a major role, platforms like Instagram or YouTube demonstrate smile transformations, aesthetic procedures, surgical procedures, and detailed step-by-step video tutorials. The portrayals mentioned above do not only help in setting patient expectations but also preserve and determine patient satisfaction levels [7]. Dental tourism, elective treatment, and aesthetic dentistry have benefitted from the persuasive appeal inherent in SM [8].

In Pakistan, the growth of SM mirrors global trends. With over 82 million active internet users in 2023, digital engagement is transforming healthcare delivery, especially in urban and semi-urban areas [9]. Yet, the impact of SM on patient experiences and satisfaction in dental care remains underexplored in this local context. Anecdotal evidence and preliminary observations suggest that patients often form expectations based on curated SM content, which may not always align with clinical realities. For instance, unrealistic aesthetic desires or misconceptions about treatment efficacy can lead to dissatisfaction [9]. At the same time, positive testimonials and before-and-after visuals may enhance trust and willingness to undergo dental procedures [9]. A few studies explored the impact of SM on dental treatment preferences [10]. However, to the best of our knowledge, studies lack exploration of the impact of SM on patient expectations and satisfaction with dental procedures.

This study aims to address this critical gap through the perspectives of dentists and patients. Hence, the primary objective of this study is to explore how social media influences patient expectations and satisfaction with dental procedures. The secondary objective is to examine the potential implications of these influences for improving and enhancing patient care in the dental field.

## Materials and methods

The study employed a qualitative phenomenological research design to explore patient and dentist perspectives regarding SM in relation to patients’ expectations and their satisfaction with dental treatments in Pakistan. A constructivist paradigm was used to explore the experience of patients and dentists to identify and analyze how SM impacted their satisfaction and expectations in the dental care context.

Fieldwork included interviews with patients using a semi-structured interview guide and focus group discussion (FGD) guide with the dentists (Appendix A, B). These tools were developed following the framework outlined by Kallio et al. (2016), ensuring a systematic approach [11]. A thorough review of the literature ensured that the questions were relevant, specific, and comprehensive. The questions for the semi-structured interviews and the FGD were pilot-tested by four participants, refined by the authors, and discussed with field experts in the contexts of content relevance, clarity, and suitability. Field testing also facilitated the utilization of the guides in that they were understandable as well as relevant to the objectives of the study.

Inclusion criteria for patients included if they were adults aged 18 or over, had undergone a dental procedure within the last six months, and were willing to participate in the study. Dentists that met the eligibility criteria of having a valid license and had at least two years of clinical practice. The exclusion criteria included patients less than 18 years of age, those who had not been through any dental procedure within the last six months, and patients not actively using SM. The dentists who lacked a valid license or had clinical experience of less than two years and were not familiar with SM were also excluded.

Semi-structured interviews having open-ended questions with patients were conducted face-to-face. Each interview lasted 19 to 31 minutes and focused on understanding how SM has shaped their typical dental expectations, perceptions, and satisfaction. Organized in small groups of eight to 10 people, there were three focus group discussions with dentists. The 58- to 73-minute discussions covered their thoughts on social media’s impact on patients’ behavior, expectations, and satisfaction, as well as communication. The open-ended nature of the questions allowed for in-depth exploration of these themes. All interviews and discussions were audio recorded with participant consent and transcribed verbatim and anonymized for confidentiality. Member checking was conducted by sharing transcripts through WhatsApp and via courier with participants to ensure the accuracy and credibility of the work.

The patterns and themes were inductively identified simultaneously with data collection and analysis through Braun and Clarke thematic analysis [12]. The data was coded and grouped into categories over repeated readings of transcripts. Data was coded twice independently by two researchers to increase reliability. Key themes were extracted and illustrated with representative quotes providing context to the findings.

**Figure 1:**
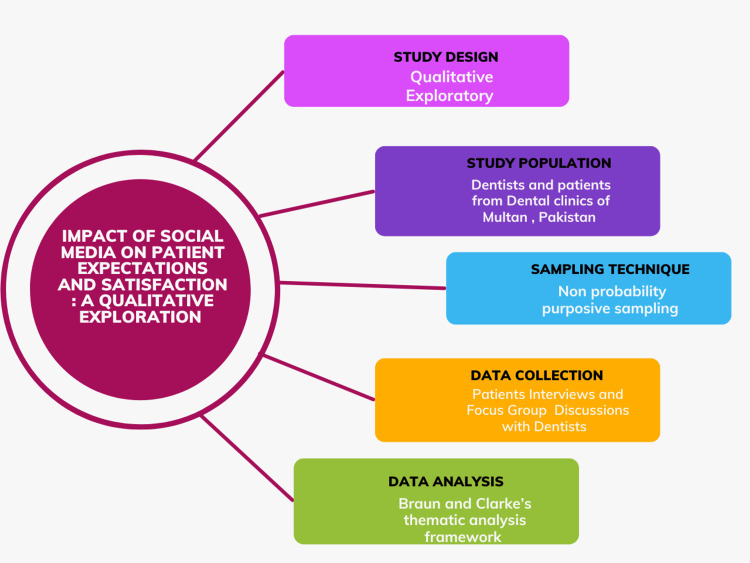
Methodology flowchart. Media element via canva.com (Canva).

Ethical approval was applied for and obtained from the Institutional Review Board (IRB) of Bakhtawar Amin Dental College and Hospital (259/23/COD, dated July 6, 2023). Participants provided written informed consent after being fully briefed about the study’s purpose, procedures, and their rights, including the right to withdraw at any stage without any consequences. Confidentiality and anonymity were maintained by using pseudonyms and securely storing all collected data with restricted access. The study followed the Consolidated Criteria for Reporting Qualitative Research (COREQ) to ensure methodological rigor (Figure 1) and transparency throughout the research process [13].

## Results

The participants comprised patients and dentists. Table 1 lists the participants' demographic details, including 10 dental consultants and 17 patients. The patient group had eight female individuals (47.1%) and nine male individuals (52.9%). The consultant's FGD consisted of four female individuals (40%) and six male individuals (60%). The age was distributed in the patients group as five participants (29.4%) between 18-25 years, six people (35.3%) between 26-35 years, four participants (23.5%) between 36-45 years, and two (11.8%) between 46-55 years. Among the consultants, three (30%) were aged 26-35 years, five (50%) were aged 36-45 years, and two (20%) were aged 46-55 years. The socioeconomic status of the patients revealed that the majority belonged to the low-income group (n=9, 52.9%), followed by the middle-income group (n=5, 29.4%) and the high-income group (n=2, 11.8%). The consultants were distributed across specialties as follows: three (30%) were orthodontists, two each (20%) specialized in prosthodontics, oral and maxillofacial surgery, and endodontics, while one (10%) specialized in periodontics. Regarding years of experience, three consultants (30%) had one to five years of experience, four (40%) had six to 10 years, two (20%) had 11-15 years, and one (10%) had over 16 years of professional practice.

**Table 1:** Demographic characteristics of the participants.

Characteristics	Patients (n=17)	Consultants (n=10)
Gender		
Male	09	6
Female	8	4
Age range (years)		
18–25	5	0
26–35	6	3
36–45	4	5
46–55	2	2
Socioeconomic status		
Low	9	N/A
Middle	5	N/A
High	3	N/A
Specialty (consultants)		
Prosthodontics	N/A	2
Orthodontics	N/A	3
Periodontics	N/A	1
Oral and maxillofacial surgery	N/A	2
Endodontics	N/A	2
Years of experience (consultants)		
1–5 years	N/A	3
6–10 years	N/A	4
11–15 years	N/A	2
16+ years	N/A	1

The study identified four overarching themes: (1) patient expectations, (2) satisfaction with care, (3) barriers to seeking care, and (4) recommendations for improvement. Each overarching theme has three subthemes as displayed in Table 2. The findings of this study revealed significant insights into patient expectations, satisfaction, and perceptions of dental procedures, particularly in the context of SM influence. Participants' opinions vary regarding treatment results, professionalism, and dental care costs. The majority of the participants (65%) stated their expectations were unmet after dental procedures, indicating inadequacies in communication and patient education on realistic grounds; hence, most of the patients (65% of the participants) expected that dental treatments would drastically improve both functionality and aesthetics. The majority of the participants (70%) also highlighted key barriers, including cost and accessibility; participants (41%) stressed the need to offer reasonable and subsidized dental care options.

**Table 2:** Patients perspectives and experiences regarding social media in dentistry. SM: social media; M: male; F: female; R: respondent; 1-17: respondent index; 18-55: age range.

Themes	Subthemes	Representative quotations
High outcome expectations	Treatment outcomes	"I expected that a removable tooth would fix my problems related to chewing food and improve my appearance, but the results were quite the opposite." (M42R07)
	Consultant professionalism	"I was expecting the dentist to listen to my concerns and answer my queries in a relaxed, calm environment." (F28R03)
	Cost and accessibility	"The charges are too high for most families like mine. Dental care should be affordable, and the government should subsidize these treatments along with others." (M41R12)
Satisfaction with dental procedures	Communication	"I selected my dentist after reading a lot of reviews on Instagram, and I was happy after treatment and detailed communication of each phase by the dentist." (F24R04)
	Treatment services	"I once saw a dental clinic’s SM post showcasing perfect smile makeovers with veneers. Expecting similar results, I proceeded with treatment but was disappointed. My teeth didn’t look as flawless due to individual differences, and I wasn’t informed about long-term maintenance." (F32R09)
	Follow-up care	"After the procedure, no one contacted me to check how I was doing. I felt ignored." (M21R08)
Misconceptions and advantages	Unreal advertisements	"I saw an SM ad showing a complete smile makeover in a single visit. Excited, I consulted my dentist, who explained that achieving lasting results often requires multiple visits for proper planning, preparation, and adjustments." (M34R05)
	Awareness tool	"SM helps me understand dental procedures through visual explanations and patient testimonials. It makes it easier to find reputable dentists, compare services, and learn about new treatments." (F27R17)
	Cultural beliefs	"In our culture and region Dental issues are not taken seriously unless they become severe; however, due to digital platforms, people are now concerned about facial aesthetics." (M48R14)
Suggestions for balanced use of SM	Affordable care	"There should be options for free online screening and low-cost treatment packages for people like us." (M25R10)
	Enhanced communication	"Dentists should add detailed descriptions while posting ads and videos on social media and should avoid misleading and unrealistic pictures or procedures." (F29R11)
	Awareness campaigns	"The government or institutions should run SM campaigns to make people aware of the importance of dental health." (M24R13)

Participants (53%) highlighted that satisfaction with dental procedures was associated with quality of communication, dental services, and follow-up care. Most of the patients (59%) who selected their dentist through reviews from SM were content as the outcomes aligned with their expectations. However, dissatisfaction emerged from treatment outcomes not being up to the expected level, a lack of adequate post-treatment attention that was perceived as negligence by the patients, or unrealistic expectations due to manipulated SM advertising.

SM plays a crucial role in raising awareness about dental treatments. Participants (47%) highlighted the significance of SM in connecting and finding good consultants with ease. However, unrealistic portrayals in SM commercials created confusion and dissatisfaction among patients. Moreover, participants (41%) opined that cultural trends related to dental health are also evolving, possibly because of the adoption of digital communication.

Participants also offered recommendations to optimize the use of SM for dental care, emphasizing the need for transparency, realistic content in advertisements, and awareness campaigns. They advocated for detailed, honest descriptions of procedures and outcomes to manage expectations effectively. Additionally, suggestions included affordable care options and government-led SM campaigns to educate the public about oral health's importance.

The FGD with dentists provided valuable insights into the professional challenges, opportunities, and ethical considerations associated with SM utilization in dental practice as displayed in Table 3.

**Table 3:** Dentists perspectives and experiences regarding social media in dentistry. SM: social media.

key topics discussed	Summary	representative quotations
Professional challenges	Social media often sets unrealistic expectations, leading to patient dissatisfaction. Misinformation can cause confusion about treatments, and managing online reviews becomes a challenge. The focus on aesthetics pressures dentists to deliver perfect results.	"Now the terms Hollywood smile and smile makeovers are very common, and patients come up with pictures to make them feel like that person within days.” (C1)
Training and mentorship	Training and mentorship of young dentists regarding social media utilization is crucial. It should focus on ethical content creation, patient engagement, and professional branding.	"We lack proper mentoring systems for young professionals, so that innovation can be benefitted within ethical boundaries." (C4)
Role of institutions	Institutions play a vital role in training young dentists to effectively use social media by integrating it into the curriculum. This includes teaching digital literacy, ethical use of platforms, and patient communication through online channels.	"Institutions should focus on regular workshops and conferences for professional growth regarding SM strategies and ethical dilemmas linked to it.” (C6)
Public awareness and education	There is a strong need for campaigns to educate the public on dental health and realistic treatment outcomes. These campaigns can address misinformation, promote preventive care, and set achievable expectations.	"There is very little awareness about dental procedures; people often don’t know what to expect." (C8)
Collaborative digital partnerships	Collaborative digital media partnerships in dentistry that involve dentists partnering with online platforms, influencers, or digital marketing agencies to enhance their practice's visibility, share educational content, and engage with a broader audience can have a positive impact on society.	"We posted ads through Facebook and Instagram, and these platforms provided every minute detail of participants, and our consultation rate improved." (C10)
Ethical considerations in practice	There are multiple ethical facets, including maintaining patient privacy and obtaining informed consent for any featured content. Transparency about sponsorships or financial ties is essential. The focus should remain on providing value to patients, not prioritizing marketing over care.	"People are posting the work of others without adding credit, disclosing patient IDs; that is against the ethics." (C7)
Use of technology	The use of social media technology integration by dentists can offer significant benefits, such as improving patient education, expanding practice visibility, and fostering stronger patient-dentist relationships. However, it must be used responsibly.	"Dentists should learn basic SM tools and their appropriate use to take positive advantage." (C9)

Professional challenges emerged as a significant theme, with participants highlighting the unrealistic expectations set by SM, particularly regarding aesthetic outcomes. Misleading content and patient exposure to idealized images created pressures for dentists to deliver “perfect” results, often within impractical timelines. Additionally, managing misinformation and online reviews presented further challenges, contributing to patient dissatisfaction.

The majority of the participants (80%) believe in the need for training and mentorship regarding SM usage for young dentists. Participants (90%) also emphasized the need for structured programs for training dentists in the ethical use of SM, patient dealing, and branding the professional practices. Participants (60%) also highlighted the institutional role related to SM training courses in the curriculum. Participants also emphasized the active role of institutions by conducting workshops, conferences, and training to promote the creation of ethical content and engagement with patients through digital platforms. The majority of the participants (70%) highlighted the need for public awareness through strategic communication to counter misinformation, unrealistic treatment expectations, preventive measures, and mitigating myths. The participants (50%) also highlighted the potential benefits of digital partnerships, including with influencers, digital media channels, and marketing agencies.

These collaborations were regarded as a medium for increasing the practice’s exposure, sharing real-time procedural information, and connecting with the broader audience. Enhanced patient consultations and increased patient connectivity were perceived as the positive impacts of these partnerships.

Ethical considerations in practice were repeatedly emphasized by participants, with major concerns about patient privacy breaches, lack of informed consent for shared content, and the unethical use of others’ work without proper credit. Transparency in sponsorships and responsible content sharing were considered essential to upholding professional integrity and patient trust.

The use of the latest technology options in SM integration was discussed as a beneficial tool for patient education, expanding visibility, and fostering strong patient-dentist relationships. However, to maximize these benefits without compromising professional ethics, participants emphasized the need for responsible and informed use of SM tools.

## Discussion

This study explored the impact of SM on patient expectations and satisfaction with dental procedures in Pakistan. The findings revealed dynamics influenced by sociocultural factors, patients' misconceptions, and institutional gaps in patient education and engagement. Participants across focus groups and interviews shared multifaceted perspectives, highlighting challenges, opportunities, and recommendations for addressing the identified issues.

Patients described how SM influences their perceptions of dental treatments, often creating unrealistic expectations about procedural outcomes. This aligns with findings from a previous study conducted in India in 2023 that highlighted how SM amplifies patients' aesthetic aspirations, sometimes overshadowing clinical realities, particularly in cosmetic and restorative dentistry [14]. Participants in the study reported a lack of structured communication to manage expectations and high-cost treatments with a lack of relief from the government level; however, the findings were contrasting from another study conducted in 2018 that highlighted sound insurance plans, along with high budget allocations in European countries, mitigated such challenges [15]. Participants reported that their satisfaction levels were tied to clear communication, quality care, and post-treatment follow-up, with gaps in these areas contributing to dissatisfaction and mistrust. The findings were complemented by another study conducted in 2021 that highlighted communication, quality care, and post-treatment follow-up significantly improved patient satisfaction [16].

The findings underscored the lack of awareness and educational initiatives to inform patients about realistic outcomes and dental procedures. These observations are also consistent with the aforementioned study, which emphasizes the critical role of patient education in managing expectations [16]. Participants also reported misuse of SM by posting incomplete and unreal information that often misled them. Similar findings were reported by another study conducted in 2019 that highlighted SM use in dentistry can lead to ethical violations and misleading information that may harm patients [17].

Socioeconomic disparities emerged as significant barriers to seeking dental care. Patients from low-income backgrounds suggested the provision of affordable treatment options. These barriers were compounded by accessibility challenges to dental care, and participants recommended community-based awareness programs for local communities. The findings aligned with broader healthcare research outcomes that outlined financial and geographic constraints as key barriers to healthcare [18].

Participants highlighted the role of SM both as a facilitator of information and as a creator of misinformation as well. Although it facilitated awareness of dental procedures and provided dentists and patients with a collaborative platform for connection, the exaggerated claims and deceptive SM advertisements created unrealistic expectations. Participants emphasized that these challenges should be addressed by the ethical and transparent use of SM. Findings also highlighted the role of SM in creating a cultural shift toward aesthetic and preventive dental procedures. However, the findings were inconsistent with the previous literature from Western countries, where there are health advertising regulatory frameworks for digital platforms, and the lack of such regulations in developing countries like Pakistan poses challenges for transparent and ethical dental practices [19].

The major challenge dentists encountered was the unrealistic patient expectations based on hyped SM marketing ads. It was also challenging for dental professionals to complete the treatment plans within the short expected timeframe for patients. A study in the United Kingdom in 2018, a country with a high SM penetration, also reported similar challenges [20]. Participants suggested structured training programs for dentists to manage their clinical practices through social media successfully yet within ethical boundaries. A study conducted in India in 2024 also emphasized structured training of dentists for effective and ethical use of digital platforms [21].

Participants also emphasized that institutions should play their role by providing training and reforming the curriculum to include recent technological advancements, including SM, to equip young dental professionals with the necessary knowledge and skills to utilize these platforms ethically and effectively. The findings are consistent with previous studies that highlighted the need for incorporating SM guidelines in curricula [22,23]. Participants also suggested structured patient education programs, faculty training, and comprehensive public awareness campaigns. These initiatives can provide patients the relevant information, enhance the skills of dental professionals, and improve community awareness regarding dental health. The findings are in line with a review published in 2021 that also highlighted that dental schools should include social media awareness discussions in their curriculum and faculty development plans to address the issues related to SM use by dental professionals [24].

This study highlighted a context-specific understanding of the effect of SM on patient expectations and satisfaction in the unique socio-cultural and economic setting of Pakistan, an understudied setting in dental healthcare research. The study employed a combination of semi-structured interviews and FGDs to allow for in-depth exploration of the perspectives of patients and dentists. The credibility and reliability of the results were enhanced through the use of rigorous quality assurance protocols (i.e., peer debriefing and member checking). In addition, the study provides practical recommendations, including public awareness campaigns, ethical utilization of SM, and improved communication practices, which have considerable implications for policy and practice.

Limitations

The geographic focus on Multan may hamper the generalisability of the findings to other regions with different sociocultural or economic dynamics. Due to the reliance on self-reported data, it may result in data bias, including recall bias or social desirability bias, and may not provide a true representation of the respondents. Although the sample size was sufficient for sampling in qualitative research, it may not represent the full range of experiences. One potential limitation of this study is the variability in how participants engage with social media, which may influence their perceptions and expectations. Different levels of exposure and types of content could lead to varying impacts on patient satisfaction and treatment expectations. Moreover, due to the cross-sectional nature of the study design, it was not possible to assess the evolving SM impact on patient expectations and satisfaction over time.

Although the study primarily focuses on dental clinics in Multan, Pakistan, its findings hold wider significance. This research can inform future initiatives aimed at improving patient engagement through tailored communication strategies that address the influence of social media. Additionally, the insights gained can guide the development of educational programs for both patients and healthcare providers, fostering a more informed and realistic understanding of dental procedures. By leveraging the findings, new approaches in patient treatments can be implemented, particularly in managing patient expectations and enhancing satisfaction through better use of digital platforms.

## Conclusions

The study revealed that SM had played a significant part in shaping patient expectations and satisfaction with dental care in Pakistan, considering its dual role as a channel for providing access to valuable information and misinformation. Findings highlighted that most of the patients developed unrealistic expectations of treatment outcomes drawn from idealized publicity images online that ultimately led to disappointment due to differences in results based on practical options available. The study findings also pointed out that the majority of dentists faced difficulty in managing hyped expectations of patients. Additionally, the study also highlighted a gap in patients’ education and awareness about dental treatments. The study also offered recommendations that included improved communication strategies using SM and institutional awareness campaigns using the technological features of SM. Furthermore, the findings also highlighted the need for transparent and realistic SM content, public awareness campaigns, and training programs regarding effective ethical digital engagement by dentists. The findings also suggested that the curricula need revision to include ethical aspects of SM usage as well as technology incorporation in practice management. The findings also highlighted that government funding and the provision of subsidized dental treatment to non-affordable patients will enhance their access to care. By making these changes, SM platforms have the potential to manage patient awareness, expectations, and satisfaction.

## Appendices

Appendix A 

**Table 4:** Semi-structured interview guide for patients.

Question number	Interview questions
1	Can you describe your experience with social media in relation to dental procedures?
2	How has social media shaped your expectations about dental treatments?
3	Have your expectations ever been unmet after undergoing a dental procedure?
4	What benefits do you think social media provides in understanding dental procedures?
5	What challenges or misconceptions have you faced when relying on social media for dental advice?
6	How do you feel about the way your dentist communicates about procedures and outcomes?
7	What improvements would you suggest for communication between patients and dentists?
8	Is there anything else you would like to share about your experiences with social media and dental treatments?

Appendix B 

**Table 5:** Focus group discussion guide for dentists.

Question number	Interview questions
1	How do you perceive the influence of social media on patient expectations regarding dental procedures?
2	What are the key challenges you face in managing patients whose expectations are shaped by social media?
3	How do you address misinformation or unrealistic demands from patients?
4	How do you typically communicate risks, benefits, and realistic outcomes to patients?
5	What role do you think social media should play in patient education?
6	What strategies would you suggest to align patient expectations with clinical realities?
7	How can social media be leveraged to educate patients about realistic outcomes and preventive care?
8	What additional training or institutional support do dentists need to address challenges posed by social media?
9	Is there anything else you would like to discuss about the role of social media in dental care?
